# Supplementary material for: Charting brain growth and aging at high spatial precision
Source: eLife. 2022 Feb 1;11:e72904. doi: 10.7554/eLife.72904 (PMC8828052; doi:10.7554/eLife.72904)
Supplement: Supplementary file 1. [file elife-72904-supp1.docx]

|  | **Train** |  |  | **Control**  **Test** |  |  | **Patient**  **Test** |  |  |
| --- | --- | --- | --- | --- | --- | --- | --- | --- | --- |
| **Site** | N | Sex (F/M) | Age (m, s.d) | N | Sex  (F/M) | Age  (m, s.d) | N | Sex (F/M) | Age  (m, s.d) |
| ABCD_01 | 194 | 100/94 | 9.95, 0.62 | 194 | 99/95 | 9.85, 0.63 |  |  |  |
| ABCD_02 | 271 | 127/144 | 10.12, 0.6 | 271 | 125/146 | 10.03, 0.64 |  |  |  |
| ABCD_03 | 285 | 131/154 | 9.86, 0.62 | 284 | 137/147 | 9.9, 0.62 |  |  |  |
| ABCD_04 | 315 | 149/166 | 9.81, 0.64 | 316 | 158/158 | 9.82, 0.65 |  |  |  |
| ABCD_05 | 172 | 91/81 | 9.9, 0.65 | 173 | 87/86 | 9.89, 0.62 |  |  |  |
| ABCD_06 | 282 | 142/140 | 9.96, 0.59 | 282 | 144/138 | 9.93, 0.59 |  |  |  |
| ABCD_07 | 162 | 74/88 | 9.87, 0.63 | 163 | 79/84 | 9.87, 0.62 |  |  |  |
| ABCD_08 | 168 | 70/98 | 10, 0.62 | 168 | 90/78 | 9.91, 0.62 |  |  |  |
| ABCD_09 | 204 | 107/97 | 9.95, 0.63 | 203 | 93/110 | 9.98, 0.59 |  |  |  |
| ABCD_10 | 287 | 135/152 | 9.85, 0.63 | 288 | 145/143 | 9.87, 0.62 |  |  |  |
| ABCD_11 | 207 | 108/99 | 9.84, 0.62 | 207 | 98/109 | 9.79, 0.64 |  |  |  |
| ABCD_12 | 81 | 33/48 | 9.89, 0.56 | 80 | 44/36 | 9.87, 0.62 |  |  |  |
| ABCD_13 | 277 | 146/131 | 9.81, 0.58 | 278 | 131/147 | 9.82, 0.61 |  |  |  |
| ABCD_14 | 292 | 131/161 | 10.17, 0.56 | 291 | 136/155 | 10.22, 0.57 |  |  |  |
| ABCD_15 | 198 | 87/111 | 9.87, 0.59 | 197 | 91/106 | 9.94, 0.62 |  |  |  |
| ABCD_16 | 460 | 205/255 | 9.91, 0.65 | 461 | 209/252 | 9.9, 0.65 |  |  |  |
| ABCD_17 | 278 | 133/145 | 9.79, 0.61 | 279 | 134/145 | 9.84, 0.64 |  |  |  |
| ABCD_18 | 171 | 82/89 | 9.92, 0.61 | 170 | 78/92 | 9.9, 0.65 |  |  |  |
| ABCD_19 | 267 | 130/137 | 10.04, 0.55 | 267 | 142/125 | 10.08, 0.54 |  |  |  |
| ABCD_20 | 320 | 162/158 | 10.05, 0.5 | 320 | 158/162 | 10.07, 0.48 |  |  |  |
| ABCD_21 | 246 | 104/142 | 9.92, 0.65 | 245 | 118/127 | 9.91, 0.6 |  |  |  |
| ABIDE_GU | 27 | 11/16 | 10.57, 1.68 | 27 | 16/11 | 10.29, 1.75 | 47 | 8/39 | 10.94, 1.53 |
| ABIDE_KKI | 93 | 32/61 | 10.35, 1.19 | 94 | 33/61 | 10.24, 1.2 | 77 | 19/58 | 10.23, 1.5 |
| ABIDE_NYU | 68 | 10/58 | 14.35, 6.01 | 67 | 18/49 | 14.47, 6.62 | 127 | 16/111 | 12.83, 6.86 |
| ABIDE_USM | 30 | 2/28 | 23.12, 6.14 | 29 | 1/28 | 20.98, 9.03 | 74 | 2/72 | 21.61, 7.78 |
| ADD200_KKI | 31 | 14/17 | 10.23, 1.28 | 30 | 13/17 | 10.27, 1.29 | 22 | 10/12 | 10.22, 1.56 |
| ADD200_NYU | 19 | 13/6 | 10.04, 2.2 | 19 | 7/12 | 10.38, 1.6 | 52 | 14/38 | 10.05, 1.67 |
| AOMIC_1000 | 464 | 235/229 | 22.87, 1.71 | 464 | 248/216 | 22.83, 1.71 |  |  |  |
| AOMIC_PIPO2 | 104 | 61/43 | 22.21, 1.91 | 105 | 59/46 | 22.16, 1.69 |  |  |  |
| ATV | 39 | 8/31 | 22.59, 1.96 | 38 | 9/29 | 22.76, 2.02 |  |  |  |
| CAMCAN | 323 | 167/156 | 55.11, 19.37 | 324 | 162/162 | 53.28, 17.7 |  |  |  |
| CIN | 33 | 11/22 | 48.64, 15.83 | 33 | 11/22 | 47.15, 18.14 |  |  |  |
| CMI-HBN_CBIC | 99 | 35/64 | 11.16, 3.73 | 100 | 38/62 | 11.24, 3.74 |  |  |  |
| CMI-HBN_RU | 188 | 57/131 | 10.37, 3.3 | 188 | 68/120 | 10.69, 3.74 |  |  |  |
| CMI-HBN_SI | 53 | 17/36 | 11.24, 3.83 | 53 | 21/32 | 10.79, 3.64 |  |  |  |
| CNP-35343.0 | 45 | 23/22 | 31.91, 8.39 | 45 | 20/25 | 31.98, 9.48 | 63 | 30/33 | 35.33, 9.75 |
| CNP-35426.0 | 10 | 6/4 | 28.1, 7.17 | 10 | 5/5 | 30.9, 8.75 | 59 | 15/44 | 34.63, 9.35 |
| COI | 62 | 39/23 | 51.92, 14.31 | 62 | 39/23 | 51.81, 12.64 | 69 | 40/29 | 45.07, 12.62 |
| delta | 24 | 10/14 | 50.17, 9.27 | 25 | 8/17 | 50.72, 8.44 | 111 | 48/63 | 45.23, 13.18 |
| ON_ds001734 | 54 | 28/26 | 25.43, 3.75 | 54 | 32/22 | 25.67, 3.45 |  |  |  |
| ON_ds002236 | 43 | 18/25 | 10.99, 1.75 | 43 | 20/23 | 11.99, 2.21 |  |  |  |
| ON_ds002330 | 33 | 19/14 | 26.52, 4.15 | 33 | 18/15 | 26.73, 4.52 |  |  |  |
| ON_ds002345 | 104 | 65/39 | 21.61, 4.12 | 103 | 66/37 | 21.79, 5.26 |  |  |  |
| ON_ds002731 | 29 | 19/10 | 21.21, 1.57 | 30 | 9/21 | 21.3, 1.34 |  |  |  |
| ON_ds002837 | 43 | 24/19 | 27.23, 10.63 | 43 | 18/25 | 26.23, 9.55 |  |  |  |
| HCP_A_MGH | 85 | 45/40 | 61.41, 16.18 | 86 | 41/45 | 58.13, 14.79 |  |  |  |
| HCP_A_UCLA | 62 | 36/26 | 51.77, 11.36 | 62 | 35/27 | 54.89, 14.03 |  |  |  |
| HCP_A_UM | 102 | 67/35 | 63.71, 16.77 | 102 | 53/49 | 59.48, 15.63 |  |  |  |
| HCP_A_WU | 89 | 57/32 | 59.79, 14.34 | 89 | 55/34 | 57.89, 12.54 |  |  |  |
| HCP_D_MGH | 108 | 57/51 | 14.4, 3.94 | 108 | 52/56 | 13.17, 3.72 |  |  |  |
| HCP_D_UCLA | 63 | 33/30 | 14.3, 3.63 | 64 | 29/35 | 13.98, 4.04 |  |  |  |
| HCP_D_UM | 78 | 41/37 | 13.71, 3.69 | 78 | 44/34 | 12.82, 3.55 |  |  |  |
| HCP_D_WU | 77 | 36/41 | 14.21, 4.25 | 77 | 39/38 | 13.73, 3.46 |  |  |  |
| HCP_EP_BWH | 4 | 1/3 | 21.92, 2.07 | 4 | 1/3 | 24.27, 4.28 | 23 | 8/15 | 22.39, 4.26 |
| HCP_EP_IU | 12 | 5/7 | 24.76, 4.21 | 13 | 6/7 | 23.12, 3.27 | 59 | 22/37 | 22.97, 3.84 |
| HCP_EP_McL | 7 | 2/5 | 26.49, 4.29 | 6 | 2/4 | 23.6, 2.33 | 31 | 15/16 | 23.56, 3.46 |
| HCP_EP_MGH | 6 | 1/5 | 26.89, 4.1 | 5 | 2/3 | 28.48, 5.97 | 10 | 3/7 | 20.32, 2.99 |
| HCP_YA | 557 | 309/248 | 28.76, 3.73 | 556 | 297/259 | 28.85, 3.66 |  |  |  |
| HKH | 15 | 7/8 | 46.27, 7.69 | 14 | 10/4 | 44.5, 11.42 | 33 | 13/20 | 44.82, 11.47 |
| HRC | 25 | 19/6 | 45.88, 12.74 | 24 | 17/7 | 37.33, 8.7 | 16 | 10/6 | 40.5, 11.48 |
| HUH | 33 | 18/15 | 38.24, 13.66 | 34 | 20/14 | 31.35, 11.45 | 57 | 25/32 | 43.33, 12.18 |
| IXI | 279 | 157/122 | 47.9, 16.57 | 279 | 156/123 | 49.53, 16.39 |  |  |  |
| KCL | 20 | 13/7 | 34.65, 14.13 | 21 | 12/9 | 33.62, 13.44 | 104 | 75/29 | 31.44, 11.86 |
| KTT | 64 | 18/46 | 30.77, 9.7 | 64 | 23/41 | 31.3, 8.88 | 47 | 21/26 | 37.89, 9.79 |
| KUT | 79 | 31/48 | 36.91, 13.35 | 80 | 35/45 | 36.11, 13.89 | 61 | 30/31 | 41.7, 11.18 |
| NKI-RS | 241 | 145/96 | 42.63, 21.75 | 241 | 66/175 | 42.63, 20.67 |  |  |  |
| Oasis2 | 93 | 61/32 | 75.92, 7.3 | 92 | 163/-71 | 77.86, 8.79 |  |  |  |
| Oasis3 | 776 | 312/464 | 69.41, 9.01 | 776 | 319/457 | 70.16, 9 |  |  |  |
| PNC | 689 | 355/334 | 14.16, 3.48 | 689 | 8/681 | 14.28, 3.54 |  |  |  |
| SWA | 50 | 7/43 | 27.34, 6.77 | 50 | 346/-296 | 29.62, 8.79 | 134 | 19/115 | 33.62, 8.73 |
| SWU_SLIM_ses1 | 274 | 162/112 | 20.07, 1.3 | 274 | 156/118 | 20.08, 1.25 |  |  |  |
| TOP | 146 | 66/80 | 34.25, 9.68 | 146 | 68/78 | 34.9, 9.43 | 531 | 254/277 | 32.42, 10.45 |
| UCDavis | 69 | 34/35 | 3.16, 0.57 | 67 | 28/39 | 3.09, 0.55 |  |  |  |
| ukb-11025.0 | 12493 | 6506/5987 | 63, 7.51 | 12493 | 6438/6055 | 62.98, 7.52 |  |  |  |
| ukb-11027.0 | 4986 | 2731/2255 | 64.35, 7.38 | 4986 | 2707/2279 | 64.44, 7.47 |  |  |  |
| UMich_CWS | 14 | 8/6 | 5.23, 1.15 | 15 | 9/6 | 5.44, 1.16 |  |  |  |
| UMich_IMPs | 107 | 59/48 | 12.91, 3.56 | 107 | 59/48 | 12.83, 3.29 |  |  |  |
| UMich_MLS | 79 | 31/48 | 19.94, 1.52 | 78 | 26/52 | 20.14, 1.5 |  |  |  |
| UMich_MTwins | 300 | 142/158 | 14.28, 1.97 | 300 | 134/166 | 14.28, 2.13 |  |  |  |
| UMich_SAD | 57 | 23/34 | 24.02, 4.75 | 57 | 20/37 | 26.56, 9.02 |  |  |  |
| UMich_SZG | 23 | 10/13 | 32.09, 11.09 | 22 | 12/10 | 31.64, 8.63 | 70 | 36/34 | 32.77, 9.77 |
| UTO | 101 | 57/44 | 35.01, 17.31 | 101 | 49/52 | 35.39, 16.53 | 108 | 39/69 | 36.1, 11.5 |
