# Supplementary material for: Charting brain growth and aging at high spatial precision
Source: eLife. 2022 Feb 1;11:e72904. doi: 10.7554/eLife.72904 (PMC8828052; doi:10.7554/eLife.72904)
Supplement: Supplementary file 2. [file elife-72904-supp2.docx]

|  | **Train** |  |  | **Test** |  |  |
| --- | --- | --- | --- | --- | --- | --- |
| **Site** | N | Sex (F/M) | Age (m, s.d) | N | Sex (F/M) | Age (m, s.d) |
| ABCD_01 | 180 | 96/84 | 9.88, 0.62 | 179 | 85/94 | 9.93, 0.63 |
| ABCD_02 | 267 | 126/141 | 10.12, 0.6 | 266 | 121/145 | 10.02, 0.64 |
| ABCD_03 | 282 | 138/144 | 9.87, 0.61 | 282 | 127/155 | 9.9, 0.62 |
| ABCD_04 | 275 | 131/144 | 9.87, 0.63 | 276 | 139/137 | 9.79, 0.66 |
| ABCD_05 | 169 | 86/83 | 9.94, 0.65 | 170 | 88/82 | 9.86, 0.6 |
| ABCD_06 | 280 | 140/140 | 9.97, 0.59 | 279 | 143/136 | 9.93, 0.59 |
| ABCD_07 | 160 | 69/91 | 9.88, 0.6 | 160 | 82/78 | 9.85, 0.65 |
| ABCD_08 | 132 | 55/77 | 9.98, 0.63 | 131 | 68/63 | 10.01, 0.6 |
| ABCD_09 | 197 | 98/99 | 9.96, 0.6 | 197 | 99/98 | 9.97, 0.63 |
| ABCD_10 | 248 | 114/134 | 9.9, 0.63 | 249 | 127/122 | 9.83, 0.61 |
| ABCD_11 | 203 | 89/114 | 9.81, 0.63 | 203 | 112/91 | 9.82, 0.63 |
| ABCD_12 | 79 | 38/41 | 9.97, 0.57 | 79 | 38/41 | 9.79, 0.61 |
| ABCD_13 | 240 | 121/119 | 9.83, 0.62 | 240 | 123/117 | 9.82, 0.59 |
| ABCD_14 | 285 | 116/169 | 10.2, 0.56 | 285 | 144/141 | 10.21, 0.57 |
| ABCD_15 | 188 | 93/95 | 9.88, 0.59 | 188 | 77/111 | 9.93, 0.61 |
| ABCD_16 | 459 | 196/263 | 9.9, 0.66 | 459 | 216/236 | 9.91, 0.65 |
| ABCD_17 | 264 | 135/129 | 9.78, 0.62 | 265 | 125/140 | 9.87, 0.64 |
| ABCD_18 | 139 | 60/79 | 9.99, 0.62 | 139 | 67/72 | 9.92, 0.64 |
| ABCD_19 | 254 | 128/126 | 10.11, 0.55 | 254 | 131/123 | 10.02, 0.54 |
| ABCD_20 | 314 | 161/153 | 10.08, 0.48 | 314 | 155/159 | 10.04, 0.5 |
| ABCD_21 | 239 | 112/127 | 9.93, 0.62 | 239 | 105/134 | 9.9, 0.62 |
| CAMCAN | 306 | 151/155 | 52.83, 18.39 | 307 | 164/163 | 53.56, 18.16 |
| CMI-HBN_RU | 170 | 60/110 | 11.3, 3.4 | 170 | 65/105 | 10.5, 3.7 |
| CMI-HBN_SI | 141 | 58/83 | 12.1, 3.9 | 141 | 66/75 | 11.4, 3.7 |
| CNP-35343.0 | 44 | 17/27 | 33.43, 9.5 | 43 | 25/18 | 30.53, 8.26 |
| CNP-35426.0 | 10 | 7/3 | 28.7, 6.15 | 9 | 4/5 | 29.33, 9.71 |
| delta | 24 | 10/14 | 50.92, 8.48 | 23 | 7/16 | 49.57, 9.44 |
| ON_ds001734 | 54 | 31/23 | 25.61, 3.8 | 54 | 29/25 | 25.48, 3.4 |
| ON_ds002236 | 37 | 17/20 | 11.55, 2.02 | 37 | 19/18 | 11.79, 2.17 |
| ON_ds002330 | 33 | 17/16 | 27.09, 4.24 | 33 | 20/13 | 26.15, 4.39 |
| ON_ds002345 | 104 | 69/35 | 21.61, 4.36 | 103 | 62/51 | 21.79, 5.06 |
| ON_ds002731 | 29 | 14/15 | 21.38, 1.7 | 30 | 14/16 | 21.13, 1.17 |
| ON_ds002837 | 43 | 23/20 | 26.3, 9.05 | 43 | 19/24 | 27.16, 11.06 |
| HCP_A_MGH | 85 | 48/37 | 60.03, 15.91 | 85 | 37/48 | 59.02, 14.68 |
| HCP_A_UCLA | 60 | 34/26 | 55.35, 13.24 | 61 | 37/24 | 50.91, 11.6 |
| HCP_A_UM | 101 | 60/41 | 61.78, 16.25 | 100 | 58/42 | 60.44, 15.59 |
| HCP_A_WU | 88 | 55/33 | 58.93, 13.48 | 88 | 55/33 | 58.25, 13.25 |
| HCP_D_MGH | 106 | 55/51 | 13.94, 3.79 | 106 | 52/54 | 13.74, 3.97 |
| HCP_D_UCLA | 63 | 29/34 | 13.41, 3.76 | 62 | 31/31 | 14.86, 3.85 |
| HCP_D_UM | 78 | 47/31 | 13.61, 3.64 | 78 | 38/40 | 12.93, 3.64 |
| HCP_D_WU | 76 | 33/43 | 13.61, 3.74 | 75 | 40/35 | 14.32, 3.94 |
| HCP_EP_BWH | 15 | 5/10 | 22.59, 4 | 16 | 5/11 | 22.55, 4.14 |
| HCP_EP_IU | 42 | 16/26 | 23.12, 4.02 | 41 | 17/24 | 23.43, 3.68 |
| HCP_EP_McL | 22 | 7/15 | 23.87, 2.94 | 22 | 3/8 | 24.2, 4.16 |
| HCP_EP_MGH | 10 | 3/7 | 27.56, 5.58 | 11 | 12/10 | 21.03, 2.99 |
| HCP_YA | 556 | 301/255 | 28.96, 3.66 | 556 | 305/251 | 28.65, 3.73 |
| IXI | 276 | 161/115 | 48.7, 16.25 | 277 | 151/126 | 48.76, 16.73 |
| KCL | 20 | 16/4 | 32.8, 13.23 | 20 | 9/11 | 34.75, 14.25 |
| NKI-RS | 229 | 152/77 | 43.98, 20.32 | 228 | 144/84 | 43.26, 20.56 |
| Oasis3 | 199 | 71/128 | 67.65, 10.93 | 198 | 60/138 | 68.58, 10.07 |
| PNC | 631 | 331/300 | 14.53, 3.38 | 631 | 330/301 | 14.53, 3.42 |
| TOP | 187 | 98/89 | 33.7, 9.5 | 188 | 67/121 | 33.25, 9.72 |
| ukb-11025.0 | 1867 | 987/880 | 62.21, 7.45 | 1868 | 954/914 | 62.43, 7.49 |
| ukb-11027.0 | 1117 | 592/525 | 63.24, 7.47 | 1117 | 630/487 | 63.06, 7.39 |
| UMich_CWS | 14 | 9/5 | 5.44, 1.18 | 15 | 8/7 | 5.24, 1.13 |
| UMich_IMPs | 107 | 56/51 | 12.96, 3.54 | 107 | 62/45 | 12.78, 3.3 |
| UMich_MTwins | 300 | 145/155 | 14.37, 2.04 | 300 | 131/169 | 14.19, 2.06 |
| UMich_SAD | 57 | 20/37 | 26.33, 7.65 | 57 | 23/34 | 24.25, 6.82 |
| UMich_SZG | 22 | 10/12 | 35.73, 9.82 | 23 | 12/11 | 28.17, 8.54 |
