# Supplementary material for: Charting brain growth and aging at high spatial precision
Source: eLife. 2022 Feb 1;11:e72904. doi: 10.7554/eLife.72904 (PMC8828052; doi:10.7554/eLife.72904)
Supplement: Supplementary file 3. — Data were downloaded from https://openneuro.org/ (Markiewicz et al., 2021) and are shared on GitHub along with code to transfer to demonstrate how to re-use models on new data. [file elife-72904-supp3.docx]

| **Site** | **N** | **Sex (M/F)** | **Age (m, s.d.)** |
| --- | --- | --- | --- |
| ds000115 | 99 | 59/40 | 22.9, 4.41 |
| ds000222 | 79 | 38/41 | 44.4, 20.12 |
| ds003416 | 87 | 51/36 | 6.49, 0.61 |
| ds003469 | 81 | 31/50 | 24.06, 5.31 |
| ds003653 | 87 | 22/65 | 28.7, 6.42 |
| ds003826 | 113 | 42/71 | 24.2, 3.48 |
